# Antibiotic use for *Vibrio* infections: important insights from surveillance data

**DOI:** 10.1186/s12879-015-0959-z

**Published:** 2015-06-11

**Authors:** Kam Cheong Wong, Anthony M. Brown, Georgina M. Luscombe, Shin Jie Wong, Kumara Mendis

**Affiliations:** Bathurst Rural Clinical School, School of Medicine, University of Western Sydney, PO Box 9008, Bathurst, NSW 2795 Australia; School of Rural Health, Sydney Medical School, University of Sydney, PO Box 1191, Orange, 2800 NSW Australia

**Keywords:** Vibrio infection, Vibriosis, Antibiotics, Antimicrobials, Surveillance

## Abstract

**Background:**

There is a paucity of data on the *in vivo* efficacy of antibiotics for lethal *Vibrio* species. Analyses of long-term surveillance datasets may provide insights into use of antibiotics to decrease mortality.

**Methods:**

The United States Centers for Disease Control and Prevention (CDC) Cholera and Other Vibrio Illness Surveillance (COVIS) dataset from 1990 to 2010, with 8056 records, was analysed to ascertain trends in antibiotics use and mortality.

**Results:**

Two-thirds of patients (5243) were prescribed antibiotics - quinolones (56.1 %), cephalosporins (24.1 %), tetracyclines (23.5 %), and penicillins (15.4 %). Considering all *Vibrio* species, the only class of antibiotic associated with reduced odds of mortality was quinolone (odds ratio 0.56, 95 % CI 0.46-0.67). Patients with *V. vulnificus* treated according to CDC recommendations had lower mortality (quinolone alone: 16.7 %, 95 % CI 10.2-26.1; tetracycline plus cephalosporin: 21.7 %, 16.8-27.5; no antibiotic: 51.1 %, 45.6-56.7; each p < 0.001). Cephalosporin alone was associated with higher mortality (36.8 %, 28.2-46.3). For *V. cholerae* non-O1, non-O139, mortality rates were lower for quinolone (0 %, 0–2.0) or tetracycline (4.3 %, 1.2-14.5) compared to no antibiotic (9.3 %, 6.4-13.3). For all *Vibrio* species, mortality rates increased with number of antibiotics in the treatment regimen (p < 0.001). Treatment regimens that included quinolone were associated with lower mortality rates regardless of the number of antibiotics used. The main clinical syndromes of patients with *V. vulnificus* infection were septicaemia (53.1 %) and wound infections (30.6 %). Mortality among *V. vulnificus* patients with septicaemia was significantly higher than for other clinical syndromes (p < 0.001). In a multivariate regression model, mortality in cases with *V. vulnificus* was associated with presence of pre-existing conditions (ORs ranged from 4.52 to 10.30), septicaemia (OR 2.64, 95 % CI 1.92-3.63) and no antibiotic treatment (OR 7.89, 95 % CI 3.94-15.80).

**Conclusion:**

In view of the lack of randomized control trials, surveillance data may inform treatment decisions for potentially lethal Vibriosis. Considering all *Vibrio* species, use of quinolones is associated with lower mortality and penicillin alone is not particularly effective. For the most lethal species, *V. vulnificus*, treatment that includes either quinolone or tetracycline is associated with lower mortality than cephalosporin alone. We recommend treating patients who present with a clinical syndrome suggestive of *V. vulnificus* infection with a treatment regimen that includes a quinolone.

## Background

The incidence of *Vibrio* infections, which can cause acute diarrhoea and potentially serious complications such as hypovolemic shock and septicaemia, continues to rise in the United States [1]. *Vibrio vulnificus* is the most lethal species, and there are limited data on the effectiveness of antibiotic use in *V. vulnificus* infections [2]. In 1988, the Centers for Disease Control and Prevention (CDC) in the United States established a surveillance system for human infections caused by all species of *Vibrio* known as “Cholera and Other *Vibrio* Illness Surveillance” (COVIS) [3]. This surveillance information has been used to inform healthcare providers and educate the public [4].

Clinical trials indicate that using an effective antibiotic as an adjuvant therapy to treat *V. cholerae* reduces the duration of diarrhoea [5, 6] and illness by almost 50 % in patients with moderate and severe dehydration [6]. Studies of treatment efficacy for lethal *Vibrio* species such as *V. vulnificus*, have inherent ethical difficulties and consequently there are no randomised control trials (RCT) of *V. vulnificus* in humans [2]. Animal model *in vivo* studies of antibiotic sensitivities to *Vibrio* infection may not all be applicable to humans due to differences in pharmacokinetic parameters [7]. Recommendations for *V. vulnificus* infections from a recent review are largely based on case reports or animal models [8]. The CDC provides recommendations on treatment regimens for *V. vulnificus*, *V. parahaemolyticus* and *V. cholerae* non-O1, non-O139 [4]. However only recommendations on treating *V. cholerae* are based on a human RCT [4].

Observational studies on lethal *Vibrio* infections, using established surveillance data over a long defined period of time, may provide insights about the associations between the use of antibiotics and patient outcomes. For example, Purcell *et al.* conducted a systematic review of the use of prophylactic antibiotics in the prevention of meningococcal disease and had to rely almost entirely on surveillance data to support their use [9]. Dechet and colleagues reviewed non-foodborne *Vibrio* infections using COVIS data (1997 to 2006) and concluded that the optimal antibiotic treatment for *Vibrio* infections remains unknown [10].

We have analysed COVIS data from 1990 to 2010: (a) to ascertain whether the COVIS data on antibiotic use were consistent with the CDC treatment recommendations, and (b) to determine the relationship between antibiotic treatment and mortality.

## Methods

### Study design and participants

Data were obtained from the CDC’s Enteric Diseases Epidemiology Branch from case report forms submitted to the COVIS during 1990 to 2010 [1]. COVIS data are collected using a standardised form [3]. The COVIS dataset included information on demographics, the *Vibrio* species isolated and the source of the specimen, clinical features (symptoms and signs), mortality, and pre-existing conditions and treatments during the 30 days prior to the *Vibrio* illness. The dataset also included information on whether or not the patient had an antibiotic as treatment for the *Vibrio* illness (i.e. yes, no, unknown), and if so, the name of the antibiotic/s.

Free text records of antibiotic names and some pre-existing conditions and treatments (specifically malignancy, immune disorders, proton pump inhibitors and antacids) were reviewed by authors KCW and SJW independently and classified using the Systematized Nomenclature of Medicine - Clinical Terms (SNOMED CT®) system (SNOMED Premium Version 1.0) and then reviewed jointly to reach consensus [11]. Pre-existing conditions included heart disease, diabetes, liver disease, alcoholism, malignancy, renal disease, haematological disease and immune disorders. SNOMED CT® codes were used to classify antibiotics into antimicrobial classes (e.g. quinolone, cephalosporin), antimicrobial subclasses (e.g. first, second and third generation cephalosporins) and to standardise and review pre-existing conditions and treatments (e.g. free text records of gastric surgery were reviewed and those considered irrelevant, such as hernia repair, were removed).

There were 8950 patients in the COVIS dataset, 6137 (68.6 %) of whom were recorded as having had antibiotics as treatment for their *Vibrio* illness. However, the specific name of the antibiotic was not recorded for 894 (14.6 %). A sensitivity analysis, whereby results were compared for analyses which either included or excluded the 894 patients who had an unnamed antibiotic for the *Vibrio* illness, was performed. There were no substantial differences in terms of their epidemiological information and mortality rates, and consequently the patients without a named antibiotic were excluded from subsequent analyses, resulting in a final sample size of N = 8056.

### Statistical analysis

For the purposes of analysis, where antibiotic use was recorded in COVIS as ‘unknown’ this was recoded as ‘no’ antibiotic used. Similarly, where data were missing or recorded as ‘unknown’ for clinical signs or symptoms, pre-existing conditions or treatments, or mortality, they were recoded as ‘absent’ or ‘no’ for analysis. Based on reported symptoms, patients with *V. vulnificus* were classified into the following clinical syndrome groups: (i) septicaemia, characterised by the isolation of the organism from blood AND the presence of either fever or shock; (ii) gastroenteritis, defined as the presence of blood in stool, OR both diarrhoea and vomiting, OR any gastrointestinal symptom AND the isolation of organism from stool; (iii) wound infection, characterised by cellulitis OR bullae OR fever in the absence of septicaemia, where the organism was isolated from a wound only; or (iv) other (not meeting any of the other criteria). Where a patient met the criteria for more than one clinical syndrome, septicaemia took precedence over gastroenteritis, and gastroenteritis over a wound infection.

Skewed continuous data were reported as medians with an interquartile range (IQR). Associations between demographics, antibiotic use and mortality were explored using unadjusted odds ratios with 95 % confidence intervals. Chi-square analyses explored univariate associations between factors such as antibiotic use and mortality. The association between the total number of antibiotics used and mortality was explored using the linear-by-linear association chi-square test. A series of univariate logistic regression models were conducted on the sub-sample of patients with *V. vulnificus* to determine predictors of mortality. Variables in these regression analyses included age, gender, year of notification, number of pre-existing conditions, clinical syndrome presentation and type of antibiotic regimen (quinolone only; quinolone and another antibiotic; at least one antibiotic, but not quinolone; or no antibiotic at all). Unadjusted odds ratios (OR) and 95 % confidence intervals (CIs) were produced. A multivariate logistic regression analysis was also conducted, including all of these variables, to produce adjusted ORs and 95 % CIs.

All analyses were conducted using SPSS (version 21; IBM, 2012) and α was set at p < 0.05.

### Ethics committee approval

All data were de-identified and the CDC confirmed that ethics approval and informed consent were not applicable for this research.

## Results

**Fig. 1:**
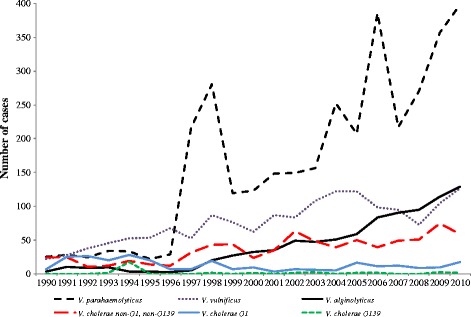
Patients with *Vibrio* infections over time, 1990 – 2010, in United States

Of the 8056 patients, the median age was 47 years (IQR 33 to 62 years; N = 7773). Over two-thirds of the patients were male (68.6 %; 5424/7905) and 98.3 % (7921/8056) of the patients had only one *Vibrio* species identified. 127 (1.6 %) patients had two species and eight (0.1 %) patients had three *Vibrio* species identified. The most commonly identified species were *V. parahaemolyticus, V. vulnificus, V. alginolyticus* and the non-O group strains of *V. cholerae* (Table 1). The proportion of fatal cases differed by *Vibrio* species, the greatest being in those with *V. vulnificus* (Table 1). Cases of *V. parahaemolyticus* increased dramatically over time, with peaks in 1998, 2004, 2006 and 2009–10, and there was a steady increase in cases of *V. alginolyticus* (Fig. 1). Peak incidence typically occurred during the summer months (June to August, 52.6 % of cases).

**Table 1:** *Vibrio* species, mortality and gender distribution; United States cases during 1990 to 2010 (N = 8056)

*Vibrio* species	Total n (%)	Mortality n (%)	Male n (%)
*V. parahaemolyticus*	3474 (43.1)	29 (0.8)	2243 (66.1)
*V. vulnificus*	1599 (19.8)	491 (30.7)	1367 (86.4)
*V. alginolyticus*	874 (10.8)	11 (1.3)	589 (69.1)
*V. cholerae* non-O1, non-O139	763 (9.5)	41 (5.4)	474 (62.9)
*V. fluvialis*	445 (5.5)	13 (2.9)	243 (56.0)
*V. cholerae* O1	269 (3.3)	4 (1.5)	137 (51.1)
*V. mimicus*	211 (2.6)	5 (2.4)	116 (55.0)
*V. hollisae* ^a^	131 (1.6)	1 (0.8)	79 (61.2)
*P. damsela* ^b^	63 (0.8)	3 (4.8)	45 (71.4)
*V. cholerae* O139	28 (0.3)	0 (0.0)	15 (57.7)
*V. furnissii*	19 (0.2)	1 (5.3)	13 (76.5)
*V. metschnikovii*	14 (0.2)	1 (7.1)	7 (53.8)
*V. cincinnatiensis*	2 (<0.1)	0 (0.0)	1 (50.0)
Species not identified	279 (3.5)	9 (3.2)	169 (61.7)
Other, no further information	28 (0.3)	0 (0.0)	16 (64.0)
Total	n/a	602 (7.5)	5424 (67.3)

N.B. some patients had more than one species isolated, so numbers total to N=8199

Proportions here are of the total patients, not total species identified

Information on gender was missing for n=151

^a^Formerly *Vibrio hollisae*, now *Grimontia hollisae* [2]

^b^Formerly *Vibrio damsela*, now *Photobacterium damsela*

**Fig. 2:**
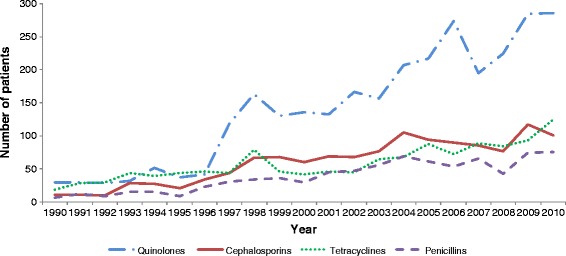
Patterns of antibiotic use in the United States for patients with *Vibrio* illness: 1990 to 2010 (N = 5243)

There were 5243 (65.1 %) patients who had an antibiotic as treatment for their *Vibrio* illness. The most commonly used antibiotics were quinolones (56.1 %), followed by cephalosporins (24.1 %), tetracyclines (23.5 %) and penicillins (15.4 %). The use of quinolones increased considerably after 1996 with peaks around 1997, 2005, and 2010, while the use of cephalosporin, tetracycline and penicillin rose only slowly from 1990 to 2010 (Fig. 2).

A total of 602 (7.5 %) patients with a *Vibrio* illness were recorded as deceased. Mortality was significantly associated with being male (OR 2.10, 95 % CI 1.70-2.59). Bullae (present in 7.3 % of cases), and shock (6.5 %) were associated with significantly increased odds of mortality (OR 8.67, 95 % CI 7.11-10.58; OR 28.49, 95 % CI 23.18-35.01 respectively). Presence of at least one of the eight pre-existing conditions (33.2 %) significantly increased risk of death (OR 16.83, 95 % CI 13.18-21.49); in particular, a history of liver disease (10.6 %; OR 18.81, 95 % CI 15.63-22.63) and alcoholism (8.9 %; OR 13.50, 95 % CI 11.19-16.27).

The association between antibiotic classes (use of at least one antibiotic of a particular class in a treatment regimen) and mortality is presented by *Vibrio* species in Table 2. Considering all patients, regardless of *Vibrio* species, the only class of antibiotic associated with reduced odds of mortality was quinolone (OR 0.56, 95 % CI 0.46-0.67). This association held for those patients with *V. vulnificus* (OR 0.58, 95 % CI 0.46-0.73), and *V. cholerae* (non-O1, non-O139) (OR 0.12, 95 % CI 0.04-0.40). Cephalosporin was associated with an increased odds of mortality (OR 2.60, 95 % CI 2.16-3.13) overall, and notably for *V. parahaemolyticus* (OR 6.41, 95 % CI 2.89-14.24). For those patients with *V. vulnificus* infection, use of the antibiotics quinolone, cephalosporin and tetracycline was associated with significantly lower mortality rate while use of penicillin was equivocal.

**Table 2:** Mortality and antibiotic use, by type of Vibrio illness (N = 8056)

*All Vibrio species*	Alive n = 7454	Deceased n = 602	Crude Odds Ratio (95% CI)
Received at least one antibiotic	65.1%	64.6%	0.98 (0.82-1.16)
At least one quinolone	37.4%	24.9%	0.56 (0.46-0.67)
At least one cephalosporin	14.5%	30.6%	2.60 (2.16-3.13)
At least one tetracycline	14.5%	25.1%	1.98 (1.63-2.40)
At least one penicillin	9.5%	15.8%	1.78 (1.41-2.25)
*V. parahaemolyticus*	n = 3445	n = 29	
Received at least one antibiotic	56.5%	65.5%	1.46 (0.68-3.15)
At least one quinolone	39.8%	37.9%	0.93 (0.44-1.97)
At least one cephalosporin	6.6%	31.0%	6·41 (2.89-14.24)
At least one tetracycline	7.3%	20.7%	3.29 (1.33-8.16)
At least one penicillin	4.6%	3.4%	0.75 (0.10-5.53)
*V. vulnificus*	n = 1108	n = 491	
Received at least one antibiotic	86.6%	68.2%	0.33 (0.26-0.43)
At least one quinolone	37.1%	25.5%	0.58 (0.46-0.73)
At least one cephalosporin	42.3%	33.2%	0.68 (0.54-0.85)
At least one tetracycline	47.5%	27.3%	0.42 (0.33-0.52)
At least one penicillin	18.5%	17.7%	0.95 (0.72-1.25)
*V. alginolyticus*	n = 863	n = 11	
Received at least one antibiotic	76.0%	36.4%	0.18 (0.05-0.62)
At least one quinolone	29.7%	18.2%	0.53 (0.11-2.46)
At least one cephalosporin	23.6%	18.2%	0.72 (0.15-3.35)
At least one tetracycline	10.0%	0.0%	n/a
At least one penicillin	19.1%	9.1%	0.42 (0.05-3.33)
*V. cholerae* non-O1, non-O139	n = 722	n = 41	
Received at least one antibiotic	66.1%	39.0%	0.33 (0.17-0.63)
At least one quinolone	39.3%	7.3%	0.12 (0.04-0.40)
At least one cephalosporin	9.4%	19.5%	2.33 (1.04-5.25)
At least one tetracycline	12.3%	19.5%	1.72 (0.77-3.85)
At least one penicillin	9.6%	7.3%	0.75 (0.23-2.48)

n/a not applicable

**Fig. 3:**
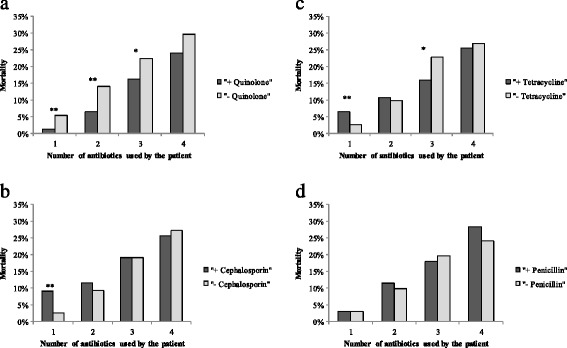
**a** Mortality by use of quinolone in treatment regimen. **b** Mortality by use of cephalosporin in treatment regimen. **c** Mortality by use of tetracycline in treatment regimen. **d** Mortality by use of penicillin in treatment regimen. ** indicates a statistically significant difference at p < 0.001, * indicates a statistically significant difference at p < 0.05. n = 3132 used one antibiotic; 1305 used two antibiotics; 652 used three antibiotics; 154 used four antibiotics

There was a statistically significant positive association between the mortality rates and the number of antibiotics used in a treatment regimen (linear by linear association = 172.90, p < 0.001). Irrespective of *Vibrio* species, a treatment regimen that included a quinolone was always associated with lower mortality rate regardless of the total number of antibiotics in the treatment regimen (Fig. 3a). Whether or not the treatment regimen included a cephalosporin, tetracycline, or penicillin was not associated consistently with the mortality rate (Fig. 3b, c, d).

Tables 3 and 4 show the mortality associated with various antibiotic regimens (including CDC recommendations) for treating *Vibrio vulnificus* and *Vibrio cholerae* non-O1, non-O139 infections. Mortality rates for *V. vulnificus* were significantly lower in those patients taking quinolone only or tetracycline combined with a third generation cephalosporin as per CDC recommendations (Table 3). Using quinolone as a reference, comparison between the CDC recommended combination (i.e. tetracycline and third generation cephalosporin) and other combinations that included quinolone, showed no statistically significant differences, except the comparison with taking a cephalosporin alone or a penicillin alone or no antibiotic (p = 0.002, p = 0.024, p < 0.001 respectively; Table 3). For *V. cholerae (non O1 and non-O139)*, only use of quinolone alone was associated with a significantly lower mortality rate (Table 4).

**Table 3:** Mortality associated with various antibiotic regimens in the treatment of *V. vulnificus* (n=1599)

	N^a^	Crude mortality % (95% CI)	Comparison with Quinolone only p value
CDC treatment recommendation^b^			
Quinolone only	14/84	16.7 (10.2-26.1)	-
Tetracycline + cephalosporin (all generations)	49/226	21.7 (16.8-27.5)	0.329
Tetracycline + 3^rd^ generation cephalosporin	39/182^c^	21.4 (16.1-28.0)	0.366
Other combinations			
Quinolone + cephalosporin (all generations)	24/98	24.5 (17.1-33.9)	0.195
Quinolone + tetracycline	15/95	15.8 (9.8-24.4)	0.874
Quinolone + cephalosporin + tetracycline	15/87	17.2 (10.7-26.5)	0.920
Other single antibiotics			
Tetracycline alone	30/145	20.7 (14.9-28.0)	0.456
Cephalosporin alone (all generations)	39/106	36.8 (28.2-46.3)	0.002
Penicillin alone	18/54	33.3 (22.2-46.6)	0.024
No antibiotic	156/305	51.1 (45.6-56.7)	<0.001

^a^n=399 patients with less common antibiotic regimens not included here, thus numbers do not total to n=1599

^b^ http://www.cdc.gov/vibrio/vibriov.html (accessed 27 June 2014): “Doxycycline (100 mg PO/IV twice a day for 7-14 days) and a third-generation cephalosporin (e.g. ceftazidime 1-2 g IV/IM every eight hours) are generally recommended. A single agent regimen with a fluoroquinolone such as levofloxacin, ciprofloxacin or gatifloxacin, has been reported to be at least as effective in an animal model as combination drug regimens with doxycycline and a cephalosporin”

^c^n=182 patients are a subset of the n=226 patients with a tetracycline combined with a cephalosporin

**Table 4:** Mortality associated with various antibiotic regimens in the treatment of *V. cholerae* non-O1, non-O139 (n=763^a^)

	N	Crude mortality % (95% CI)	Comparison with Tetracycline alone FET p value
CDC treatment recommendation ^b^			
Tetracycline alone	2/46	4.3 (1.2-14.5)	-
Other single antibiotics			
Quinolone alone	0/192	0.0 (0.0-2.0)	0.037
Penicillin alone	1/38	2.6 (0.5-13.5)	1.000
Cephalosporin alone	2/29	6.9 (1.9-22.0)	0.638
No antibiotic	25/270	9.3 (6.4-13.3)	0.395

*FET* fisher’s exact test

^a^n=188 patients with less common antibiotic regimens not included here, thus numbers do not total to n=763

^b^ http://www.cdc.gov/cholera/treatment/antibiotic-treatment.html (accessed 27 June 2014): “Antibiotic choices should be informed by local antibiotic susceptibility patterns. In most countries, doxycycline is recommended as first-line treatment for adults, while azithromycin is recommended as first-line treatment for children and pregnant women”

Considering only *V. vulnificus,* 53.1 % (839/1581) had septicaemia, 4.5 % (71) had gastroenteritis, 30.6 % (484) had a wound infection, and 11.8 % (187) had other clinical presentation. Eighteen cases were excluded because they could not be classified as information on the sample source was missing. Cases with septicaemia were more likely to fatal (40.6 %, 341/839), followed by 28.9 % (54) of those with other presentation, 28.2 % (20) of those with gastroenteritis and 14.3 % (69) of those with wound infections (p < 0.001). A series of logistic regression analyses with single predictor variables was conducted, with year of notification dichotomised into 1990 to 1996 versus 1997 to 2010 to reflect the significant increase in quinolone use from 1996 observed in Fig. 2. The crude odds of mortality amongst those with *V. vulnificus* increased significantly with the number of pre-existing conditions (see Table 5). In comparison to wound infections, all other clinical syndromes conferred increased odds of dying. Compared to quinolone alone, an antibiotic regimen without quinolone, and no antibiotic use at all were associated with increased odds of mortality. The multivariate analysis on mortality for patients with *V. vulnificus* infection showed the same patterns for number of pre-existing conditions. Gastroenteritis no longer conferred significantly greater odds of dying over wound infections, but septicaemia and other clinical syndrome did. In the adjusted model, quinolone alone remained superior in comparison to no antibiotic treatment.

**Table 5:** Predictors of death in cases of *V. vulnificus* in the United States during 1990 to 2010

	Fatal N (%)	Non-fatal N (%)	Crude OR (95% CI)	Adjusted OR (95% CI)
Age, mean (SD)	56.6 (13.5)	58.0 (17.8)	0.995 (0.989-1.001)	0.99 (0.98-0.998)
Gender				
Female	72 (15.2)	135 (12.6)	reference	reference
Male	402 (84.8)	939 (87.4)	0.80 (0.59-1.09)	0.70 (0.49-1.000)
Year of notification				
1990 – 1996	103 (21.7)	194 (18.1)	reference	reference
1997 – 2010	371 (78.3)	880 (81.9)	0.79 (0.61-1.04)	0.99 (0.73-1.34)
Pre-existing conditions				
0	41 (8.6)	397 (37.0)	reference	reference
1	107 (22.6)	277 (25.8)	3.74 (2.53-5.53)	4.52 (2.92-6.99)
2	194 (40.9)	227 (21.1)	8.28 (5.69-12.03)	10.30 (6.72-15.78)
3 or more	132 (27.8)	173 (16.1)	7.39 (4.99-10.95)	9.31 (5.93-14.62)
Clinical presentation				
Wound	68 (14.3)	406 (37.8)	reference	reference
Gastroenteritis	20 (4.2)	49 (4.6)	2.44 (1.36-4.35)	1.52 (0.77-3.01)
Septicaemia	333 (70.3)	493 (45.9)	4.03 (3.01-5.40)	2.64 (1.92-3.63)
Other	53 (11.2)	126 (11.7)	2.51 (1.67-3.79)	1.69 (1.04-2.74)
Antibiotic treatment				
Quinolone only	13 (2.7)	68 (6.3)	reference	reference
Quinolone and other/s	106 (22.4)	335 (31.2)	1.66 (0.88-3.12)	1.44 (0.73-2.84)
Antibiotic/s, not quinolone	208 (43.9)	534 (49.7)	2.04 (1.10-3.77)	1.84 (0.95-3.56)
None	147 (31.0)	137 (12.8)	5.61 (2.97-10.62)	7.89 (3.94-15.80)

## Discussion

Using this large surveillance dataset covering more than two decades, we determined that the two most important factors associated with mortality are the particular *Vibrio* species and the class of antibiotic used. Quinolone is the only class of antibiotic associated with lower mortality in all *Vibrio* species, regardless of the number of antibiotics used in a treatment regimen. In potentially lethal *V. vulnificus* infections, the use of quinolone alone or the combination of tetracycline and third generation cephalosporin had the lowest mortality which is in-line with the current CDC recommendations.

Penicillin was the least effective antibiotic for vibriosis according to our analysis. In 1984, the USA National Institutes of Health recommended use of penicillin or tetracycline to treat *V. vulnificus* based on *in vitro* sensitivity studies [12]. Morris cautioned that *in vitro* data can be misleading, and recommended against using penicillin as a single antibiotic to treat *V. vulnificus* infections in humans [13]. In 2002, Tang conducted a study on mice and reported that quinolones as single antibiotics were as effective as cefotaxime-minocycline (third generation cephalosporin and tetracycline) in combination [7]. However Tang cautioned that results of animal models may not be applicable to humans due to differences in pharmacokinetic parameters [7]. Our findings support Morris’s caution against penicillin as a single antibiotic (i.e. penicillin was not associated with reduced mortality) and substantiate Tang’s previous findings in animals (i.e. third generation cephalosporin and tetracycline in combination are more effective). More recently Shaw and colleagues evaluated the antimicrobial susceptibility of *V. vulnificus* recovered from two commercial environmental areas and found that *V. vulnificus* demonstrated resistance to penicillin [14], which may further confirm the inefficacy of penicillin.

For *V. cholerae* (non-O1, non-O139), quinolone was the only class of antibiotic associated with lower mortality rate. For *V. parahaemolyticus,* quinolone and penicillin appeared to have equivocal odds ratios for mortality; while cephalosporin and tetracycline were associated with higher mortality. However, the total number of deaths from *V. cholerae* was 41 and for *V. parahaemolyticus* was 29, so conclusions are limited.

Generally, the mortality rate increased along with the number of antibiotics used in the treatment regimen. We postulated that seriously ill patients were given more than one antibiotic and were associated with increased number of antibiotics in their treatment; hence, their mortality rate was higher possibly because they were sicker instead of due to the larger number of antibiotics used. We found that quinolone was the only antibiotic associated with reduced mortality rate regardless of the number of antibiotics in the patient’s regimen.

We have shown an increase in use of quinolone after 1996 but this may be a reflection of increased use in the wider community. Linder and colleagues have reported a three-fold increase in prescribing quinolone between 1995 and 2005 in the United States adult population [15]. This increase in quinolone prescription may not necessarily reflect increased recognition of the efficacy of this antibiotic amongst prescribers or the promulgation of treatment guidelines. It is likely that marketing, advertising and provision of sample antibiotics might have partly contributed to the increase in prescribing of newer antibiotics [15–17].

We found associations between liver disease, alcoholism and previous ill health and mortality with all species. For *V. vulnificus* the number of pre-existing conditions was associated with increased odds of mortality in both unadjusted and adjusted models. Others have found that patients with liver disease or alcoholism are at higher risk of *V. vulnificus* infection [10, 18, 19]. This may be because *V. vulnificus* uses transferrin-bound iron, which is usually abundant in these patients, for growth [18, 20]. Another hypothesis about the increase mortality in the presence of liver disease is that the shunting of portal blood containing V. *vulnificus* infection around a diseased liver may lead to septicaemia [21]. The main clinical syndromes in patients with *V. vulnificus* infection were septicaemia and wound infections. Mortality among *V. vulnificus* patients with septicaemia was significantly higher than for other clinical syndromes. A treatment regimen that included quinolone was associated with lower mortality compared with cephalosporin alone or penicillin alone or no antibiotic at all. We recommend that patients who present with a clinical syndrome suggestive of *V. vulnificus* infection be treated with a regimen that includes a quinolone.

There are a number of limitations to this study that relate to the underlying surveillance data collection. For example, non-cholera vibriosis only became nationally notifiable in USA from 2007 onwards [22]. Several pertinent details regarding use of antibiotics were not recorded systematically such as timing or order of antibiotic use. There were 894 patients who had an unnamed antibiotic for the *Vibrio* infection. Cephalosporins could not be categorised into the four generations due to the small subgroup size. This may have contributed to the paradoxical observation in Table 2 where a treatment regimen that had included a cephalosporin was associated with increased mortality rate in *V. cholera (non-O1 and non-O139)* and *V. parahaemolyticus* infections, but reduced mortality elsewhere. The dataset did not detail specific cause of death, but an infection such as *Vibrio* is likely to have been significant. Further characterisation of liver disease (type and severity) was not possible due to the inconsistency of these data in the COVIS dataset. Finally, it is possible that the use and effectiveness of different antibiotics reflect changes in the antibiotic sensitivities of the organism, however we are unable to explore this with the data available in the COVIS dataset.

Determining the optimal antibiotic regimen for a potentially lethal infection is difficult because randomised controlled trials may not be possible and *in vitro* or animal models may not be easily applied in patients. In this context, systematic experience from detailed surveillance data may inform treatment decisions. However information from surveillance data is only as good as the data collected. The COVIS dataset may not have included every *Vibrio* cases and reporting may have been biased towards more severe cases [22]. It is important that clinicians provide detailed and timely data to surveillance programs such as COVIS. Publication of findings from surveillance data may help encourage clinicians to provide specific data regarding antibiotic use. Surveillance authorities must be encouraged to simplify and refine the data collection tools and seek more specific information on classes of antibiotics and the time frame of their use so that the influence of these important factors can be reported and analysed.

## Conclusions

Surveillance of large numbers of affected individuals over longer periods of time appears to be a reasonable method of determining antibiotic use and outcome patterns. *Vibrio* infection remains a serious condition with significant mortality. Adjuvant antibiotic therapy in addition to basic care with fluids has an important role. The use of quinolones may reduce risk of death in patients with *V. vulnificus* and *V. cholerae* (non-O1 and non-O139). For *V. vulnificus*, which has the highest mortality rate, a treatment regimen which is in line with the CDC recommendations i.e. that includes either quinolone alone, or tetracycline and a third generation cephalosporin is associated with lower mortality. Penicillin alone is not particularly effective. We recommend treating patients who present with a clinical syndrome suggestive of *V. vulnificus* infection with a treatment regimen that includes a quinolone.
